# Quercetin Alleviates Insulin Resistance and Repairs Intestinal Barrier in *db*/*db* Mice by Modulating Gut Microbiota

**DOI:** 10.3390/nu16121870

**Published:** 2024-06-14

**Authors:** Man Yuan, Tieqiang Sun, Yuxian Zhang, Changjiang Guo, Feng Wang, Zhanxin Yao, Lixia Yu

**Affiliations:** Military Medical Sciences Academy, Beijing 100039, China; yuanmantj1996@126.com (M.Y.); suntq106@163.com (T.S.); yzz0088@163.com (Y.Z.); guocjtj@126.com (C.G.); wangfengseu@sohu.com (F.W.);

**Keywords:** quercetin, type 2 diabetes mellitus, insulin resistance, intestinal permeability, gut microbiota, intestinal metabolites

## Abstract

Type 2 diabetes mellitus (T2DM) is a chronic metabolic disease which seriously affects public health. Gut microbiota remains a dynamic balance state in healthy individuals, and its disorder may affect health status and even results in metabolic diseases. Quercetin, a natural flavonoid, has been shown to have biological activities that can be used in the prevention and treatment of metabolic diseases. This study aimed to explore the mechanism of quercetin in alleviating T2DM based on gut microbiota. *db*/*db* mice were adopted as the model for T2DM in this study. After 10 weeks of administration, quercetin could significantly decrease the levels of body weight, fasting blood glucose (FBG), serum insulin (INS), the homeostasis model assessment of insulin resistance (HOMA-IR), monocyte chemoattractant protein-1 (MCP-1), D-lactic acid (D-LA), and lipopolysaccharide (LPS) in *db*/*db* mice. 16S rRNA gene sequencing and untargeted metabolomics analysis were performed to compare the differences of gut microbiota and metabolites among the groups. The results demonstrated that quercetin decreased the abundance of Proteobacteria, *Bacteroides*, *Escherichia-Shigella* and *Escherichia_coli*. Moreover, metabolomics analysis showed that the levels of L-Dopa and S-Adenosyl-L-methionine (SAM) were significantly increased, but 3-Methoxytyramine (3-MET), L-Aspartic acid, L-Glutamic acid, and Androstenedione were significantly decreased under quercetin intervention. Taken together, quercetin could exert its hypoglycemic effect, alleviate insulin resistance, repair the intestinal barrier, remodel the intestinal microbiota, and alter the metabolites of *db*/*db* mice.

## 1. Introduction

Diabetes mellitus (DM) is a group of metabolic diseases characterized by chronic elevation of blood glucose and has become a global public health problem. According to the Diabetes Atlas (10th edition) reported by the International Diabetes Federation (IDF), about 537 million adults worldwide had DM in 2021, and the number is expected to reach 783 million by 2045. Type 2 diabetes mellitus (T2DM), accounting for more than 90% of patients with DM, is mainly characterized by insulin resistance (IR) and relative insulin deficiency and leads to various complications such as renal impairment, retinopathy, peripheral neuropathy, and gastrointestinal disorders [1,2,3]. In recent years, the prevalence of young-onset T2DM, which is associated with earlier onset of chronic complications and higher risk of early death [4,5], has been dramatically increasing [6]. Thus, the prevention and treatment of type 2 diabetes should be paid enough attention.

As yet, the molecular pathogenesis of T2DM remains unclear. It has been shown that inflammatory response and increased oxidative stress may be crucial factors in the development of T2DM [7,8]. Intestinal inflammatory damage is one of the metabolic disorders induced by diabetes [9]. Many studies have demonstrated that patients with type 2 diabetes are accompanied by disturbances of the intestinal microbiota and disruption of intestinal permeability, which can cause damage to multiple organs and lead to a number of complications [10,11,12]. On the side, there is growing evidence that gut microbiota plays an important role in T2DM. A population-based cross-sectional study found that the gut microbiota composition was already altered in individuals with prediabetes and is in strong correlation with insulin resistance status [13]. The gut microbiome has emerged as a potential new target for the prevention and management of T2DM and its associated complications [14]. Now there is growing concern about the potential anti-diabetic effects of traditional herbs and phytochemicals contained in plants.

Quercetin, a flavonoid widely found in flowers, leaves, and fruits of plants, is mainly metabolized in the gut and then absorbed by the body through the transformation of intestinal microbes [15,16]. Quercetin has been shown to have some biological functions, such as anti-inflammatory, antioxidant as well as hypoglycemic actions, which can be used in the prevention and treatment of type 2 diabetes [17,18]. Our research team carried out a cross-sectional study involving close to 20,000 individuals, which revealed a significant inverse correlation between dietary quercetin consumption and the prevalence of T2DM within the Chinese population, suggesting that adequate dietary intake of quercetin may have a beneficial role in the prevention of T2DM [19]. Recent studies have demonstrated that quercetin can help improve imbalances in the gut microbiota, promote a healthier intestinal microecological environment, and restore the structure and function of the intestinal barrier [20,21]. However, it is still unknown how quercetin affects intestinal flora and its metabolic small molecules, which in turn affects intestinal function and ultimately alleviates diabetic symptoms.

Our study aimed to investigate the effects of quercetin on the composition of the gut microbiota and its metabolites to have an impact on glucose metabolism and intestinal permeability in *db*/*db* mice. The present study may contribute to further exploring the complex interactions among quercetin, gut microbiota, and metabolites in the diabetic state, elucidating the mechanism of quercetin and providing a strong basis for quercetin in the treatment of T2DM.

## 2. Materials and Methods

### 2.1. Animals and Treatments

**Figure 1:**
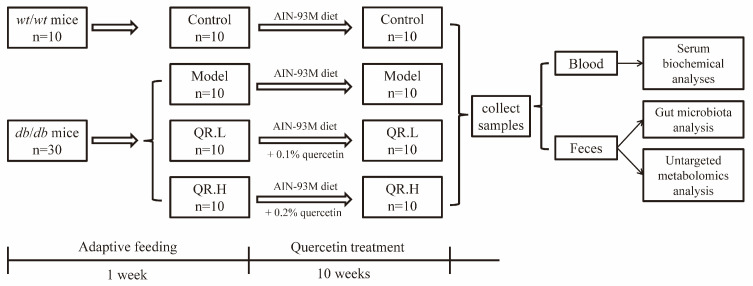
Schematic overview of experimental protocol.

The leptin receptor (Lepr) gene mutant (*db*/*db*) mice and their wild-type (*wt*/*wt*) counterparts, aged 6 weeks, were purchased from Gempharmatech Co., Ltd., (Changchou, China). Mice were placed in individual ventilated cages in groups of 5 at an ambient temperature of 22 °C–24 °C and 40–60% relative humidity with a 12 h light/dark cycle. All the mice were allowed a one-week acclimation period to adapt to the experimental environment. The standardized diet referenced the AIN-93M diet formulated by the American Institute of Nutrition [22]. Following the acclimation period, thirty *db*/*db* mice were randomly divided into three groups (*n* = 10): model group fed basic AIN-93M diet, QR.L group fed AIN-93M diet supplemented with 0.1% quercetin, and QR.H group fed AIN-93M diet supplemented with 0.2% quercetin (Sigma, St Louis, MO, USA), with the doses referenced to previous articles [23,24]. Apart from that, the *wt*/*wt* mice were fed the AIN-93M diet as a healthy control group (*n* = 10). The experiment was conducted over 10 weeks. Upon completion of the experiment, blood samples were collected via retro-orbital venous puncture, and fecal samples were aseptically assembled in aseptic cryotubes, flash-frozen in liquid nitrogen, and stored at −80 °C for subsequent analyses. The experimental protocol is shown in Figure 1.

All animal experiments conducted as part of this study were reviewed and approved by the ethics committee of the Institute of Environmental and Operational Medicine (approval number: IACUC of AMMS-04-2020-049). The experimental protocols and procedures adhered to the Regulations for the Administration of Affairs Concerning Experimental Animals (2017) and Guide for the Care and Use of Laboratory Animals (eighth edition).

### 2.2. Serum Biochemical Analyses of Glucose Metabolism

Body weights (BW), feed consumption, and fasting blood glucoses (FBG) were recorded at the same time weekly. FBG levels were measured after a 12 h fast with a glucometer (Accu-Check Performa Nano, Roche, Mannheim, Germany). Serum insulin (INS) and monocyte chemoattractant protein-1 (MCP-1) levels were measured using a Milliplex MAP Mouse adipokine magnetic bead panel kit (cat# MADKMAG-71K, Millipore, Billerica, MA, USA) and analyzed by Luminex MAGPIX^®^ (Luminex Corporation, Austin, TX, USA). The homeostasis model assessment of insulin resistance (HOMA-IR) was calculated by the formula: HOMA-IR = FBG (mmol/L) × INS (μU/mL)/22.5.

### 2.3. Serum Biomarkers of Intestinal Permeability

Serum D-lactic acid (D-LA), lipopolysaccharide (LPS) levels and diamine oxidase (DAO) activity were measured using ELISA kits supplied by ColorfulGene Biological Technology Co., Ltd., (Wuhan, China). All measurements were conducted strictly in adherence to the manufacturer’s protocols.

### 2.4. Gut Microbiota Analysis

Sequencing analysis was performed in reference to a previously published article [25]. Genomic DNA was extracted from fecal samples using the cetyltrimethylammonium bromide method. DNA concentration and purity were determined on 1% agarose gels. Then the extracted DNA samples were diluted to 1 ng/μL. The V3–V4 regions of 16S rRNA genes were amplified using primers 515F/806R with the barcode, and the PCR reactions were performed with the Phusion^®^ High-Fidelity PCR Master Mix with GC Buffer (New England Biolabs, Beverly, MA, USA). The same volume of loading buffer was mixed with the PCR products and detected by electrophoresis on 2% agarose gel. Then, the mixture PCR products were purified using the Gel Extraction Kit from the Qiagen company. Furthermore, we used the TruSeq^®^ DNA PCR-Free Sample Preparation Kit (Illumina, San Diego, CA, USA) to generate sequencing libraries, and its quality was assessed on the Qubit@ 2.0 Fluorometer and Agilent Bioanalyzer 2100 system. In the end, the samples were sequenced using an Illumina NovaSeq platform to generate paired-end reads, which were assigned to samples according to their unique barcode and truncated by cutting off the barcode and primer sequence and merged using FLASH (V1.2.7, http://ccb.jhu.edu/software/FLASH/, accessed on 28 January 2021). The same operational taxonomic units (OTUs) were defined as the sequences with ≥97% similarity. The results of sequencing were demonstrated by alpha diversity and beta diversity analysis. Differences in alpha diversity among the groups were analyzed for statistical significance using the Kruskal–Wallis test. Principal co-ordinates analysis (PCoA) was performed based on Jensen–Shannon divergence, and ANOSIM was used for statistical analysis.

### 2.5. Untargeted Metabolomics Analysis

Approximately 100 mg of fecal samples was ground under liquid nitrogen. The ground samples were then resuspended in 500 μL of 80% methanol and thoroughly vortexed for 30 s. The homogenate was incubated on ice for 10 min and then centrifuged at 15,000× *g*, 4 °C for 20 min. Some of supernatant was subsequently transferred to a new Eppendorf tube and was diluted to a final concentration containing 53% methanol. Then, the liquid was centrifuged again at 15,000× *g*, 4 °C for 20 min. Finally, the supernatant was injected into the LC-MS/MS system for analysis.

UHPLC-MS/MS analyses were conducted using a Vanquish UHPLC system (Thermo Fisher, Waltham, MA, USA) coupled with an Orbitrap Q Exactive^TM^HF-X mass spectrometer (Thermo Fisher, Waltham, MA, USA). Samples were injected onto a Hypesil Gold column (100 × 2.1 mm, 1.9 μm, Thermo Fisher, Waltham, MA, USA) using a 17 min linear gradient at a flow rate of 0.2 mL/min.

The raw data files generated by UHPLC-MS/MS were processed using the Compound Discoverer 3.1 to perform peak alignment, peak picking, and quantitation for each metabolite. After that, peak intensities were normalized to the total spectral intensity. The normalized data were used to predict the molecular formula based on additive ions, molecular ion peaks and fragment ions. And then peaks were matched with the mzCloud (https://www.mzcloud.org/, accessed on 15 April 2021), mzVault, and MassList database to obtain the accurate qualitative and relative quantitative results. These metabolites were annotated using the KEGG database (https://www.genome.jp/kegg/pathway.html, accessed on 15 April 2021).

### 2.6. Statistical Analysis

SPSS 22.0 (SPSS Inc., Chicago, IL, USA) was applied to perform statistical analysis. Figures were drawn using GraphPad Prism 8.0.1 (GraphPad Software Inc., La Jolla, CA, USA) and RStudio (https://www.rstudio.com/, accessed on 21 February 2023). Data conforming to normal distribution were presented as mean ± SEM. The independent *t*-tests were performed to analyze the significant differences between the control and model group. The analysis of difference between the control and experimental groups was conducted by Dunnett’s *t*-test. ANOVA was used to analyze the significance of the differences among groups. Correlations between microbiota and biochemical indicators and metabolites were determined by Pearson correlation analysis. *p* < 0.05 was accepted as statistically significant.

## 3. Results

### 3.1. Quercetin Reduced BW, FBG, and Insulin Levels and Alleviated IR in db/db Mice

**Figure 2:**
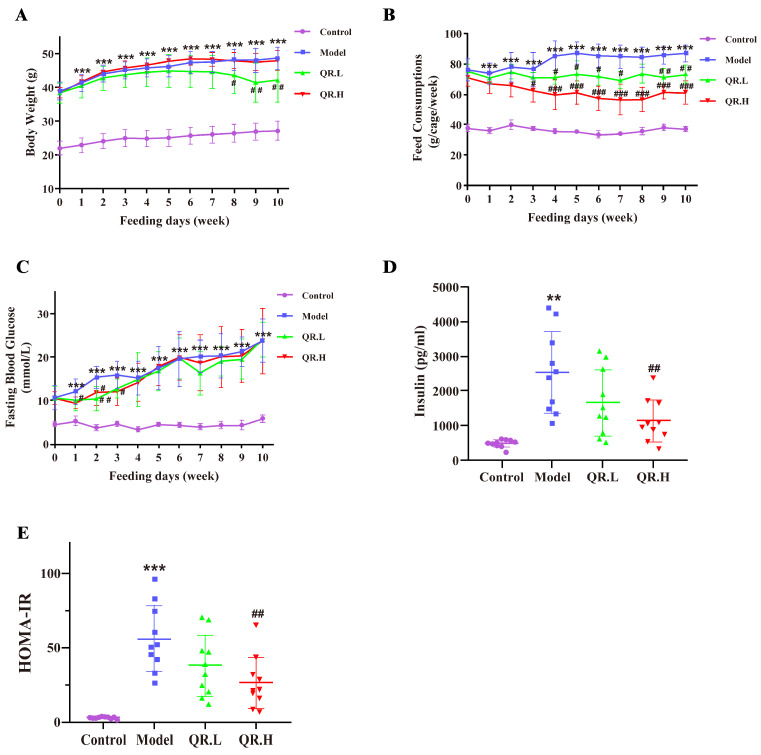
Effects of quercetin on glucose homeostasis and insulin resistance. (**A**) Body weight. (**B**) Feed consumption. (**C**) FBGs at the baseline and after 10 weeks of treatment. (**D**) Insulin levels. (**E**) The HOMA-IR index. Data are expressed as means ± SEM (*n* = 10/group). ** *p* < 0.01, *** *p* < 0.001 vs. control; ^#^ *p* < 0.05, ^##^ *p* < 0.01, ^###^ *p* < 0.001 vs. model.

During the experiment, we continuously monitored BW, feed consumptions, and FBG of each mouse. There was no significant difference in BW among the *db*/*db* mice at baseline, and the BW of *wt*/*wt* mice was consistently significantly lower than that of *db*/*db* mice. Over the course of weeks 8–10 in the intervention phase, we found that BWs in the QR.L group were statistically reduced versus the model group (*p* < 0.05) (Figure 2A). The feed consumptions of quercetin-treated groups were consistently lower than that of the model group from the third week after quercetin intervention (Figure 2B). Before the intervention, FBG levels did not differ significantly among the model, QR.L and QR.H groups, but all were elevated compared to *wt*/*wt* mice. In the first three weeks, FBG levels in the QR.H group were significantly reduced by high-dose (0.2%) quercetin treatment (*p* < 0.05). Likewise, FBG levels were significantly lower in the QR.L group than in the model group (*p* < 0.01) at the second week. However, there was no more significant difference in FBG among the groups of diabetic mice from the fourth week (Figure 2C). Insulin levels in serum were measured and HOMA-IR values were calculated at the end of the experiment. Serum insulin levels were significantly increased in the model group (*p* < 0.01) and decreased in the QR.H group treated with 0.2% quercetin (*p* < 0.01), and there was a decreased tendency of serum insulin in the QR.L group (Figure 2D). Corresponding to the results of insulin, the HOMA-IRs of the model mice were significantly higher than the *wt*/*wt* mice (*p* < 0.001), and those of the QR.H mice were significantly lower than the model mice (*p* < 0.01), which indicated that quercetin intervention could alleviate IR in *db*/*db* mice (Figure 2E).

### 3.2. Quercetin Reduced Inflammation and Repaired Intestinal Barrier in db/db Mice

**Figure 3:**
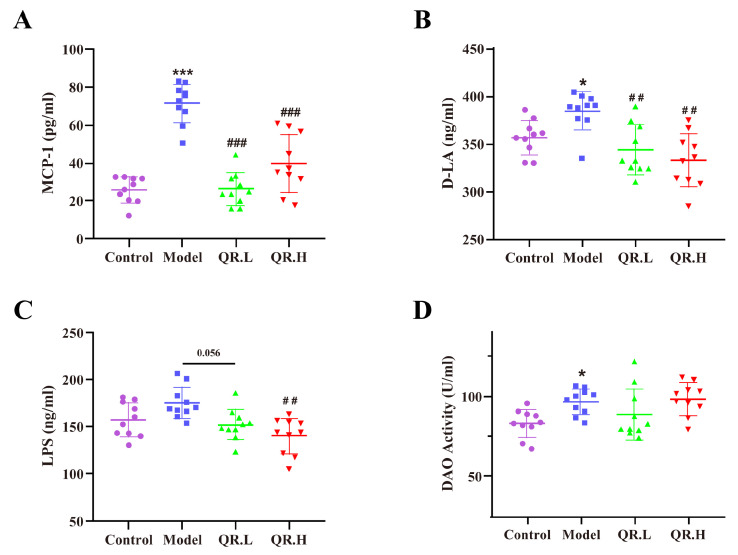
Quercetin reduced inflammation and repaired intestinal barrier. (**A**) MCP-1 levels in serum. (**B**) D-LA levels in serum. (**C**) LPS levels in serum. (**D**) DAO activity. Data are expressed as means ± SEM (*n* = 10/group). * *p* < 0.05, *** *p* < 0.001 vs. control; **^##^** *p* < 0.01, **^###^** *p* < 0.001 vs. model.

To investigate the effect of quercetin on inflammation and intestinal permeability in *db*/*db* mice, we measured an inflammatory marker MCP-1 and intestinal markers (D-LA level, LPS level, and DAO activity) in serum. As shown in Figure 3A, the levels of chemokine MCP-1 were significantly increased in the model group as compared to the control group (*p* < 0.001) but decreased after quercetin interventions in the QR.L and QR.H group (all *p* < 0.001). As a product of food fermentation in the intestine, the serum level of D-LA was significantly elevated in the model group (*p* < 0.05), while it notably declined in the QR.L and QR.H groups (*p* < 0.01) (Figure 3B). LPS could enter the bloodstream through the damaged intestine and trigger inflammation. The LPS level in the serum of *db*/*db* mice in the model group showed an elevated trend compared with the wild-type mice. As expected, a lower LPS level was found in the QR.L group (*p* = 0.056) and QR.H group (*p* < 0.01) (Figure 3C). After 10 weeks of quercetin treatment, DAO was located in the upper villi of the small intestinal mucosa, and its activity was abnormally enhanced in model group (*p* < 0.05). However, quercetin intervention had no effect on DAO enzymatic activity (Figure 3D). These results suggested that diabetic mice had an obviously inflammatory response and impaired intestinal function. Moreover, quercetin intervention could reduce inflammation and repair the intestinal barrier in *db*/*db* mice.

### 3.3. Quercetin Altered the Composition of the Fecal Microbial Community in db/db Mice

In order to find out the effect of quercetin treatment on the gut microbiota of *db*/*db* mice, we performed 16S rRNA sequencing (V3~V4 region) of the fecal samples and 1,147,709 reads for a total of 20 samples were generated. The rarefaction curve, rank abundance curve, and species accumulation boxplot of all samples revealed that the sequencing depth was sufficient and the sample size was effective (Supplementary Figure S1). Sequences with 97% identity were classified as the same operational taxonomic units (OTUs), and a total of 1098 OTUs were identified. After excluding those OTUs with less than one count, 880 effective OTUs were finally used for alpha and beta diversity analysis.

**Figure 4:**
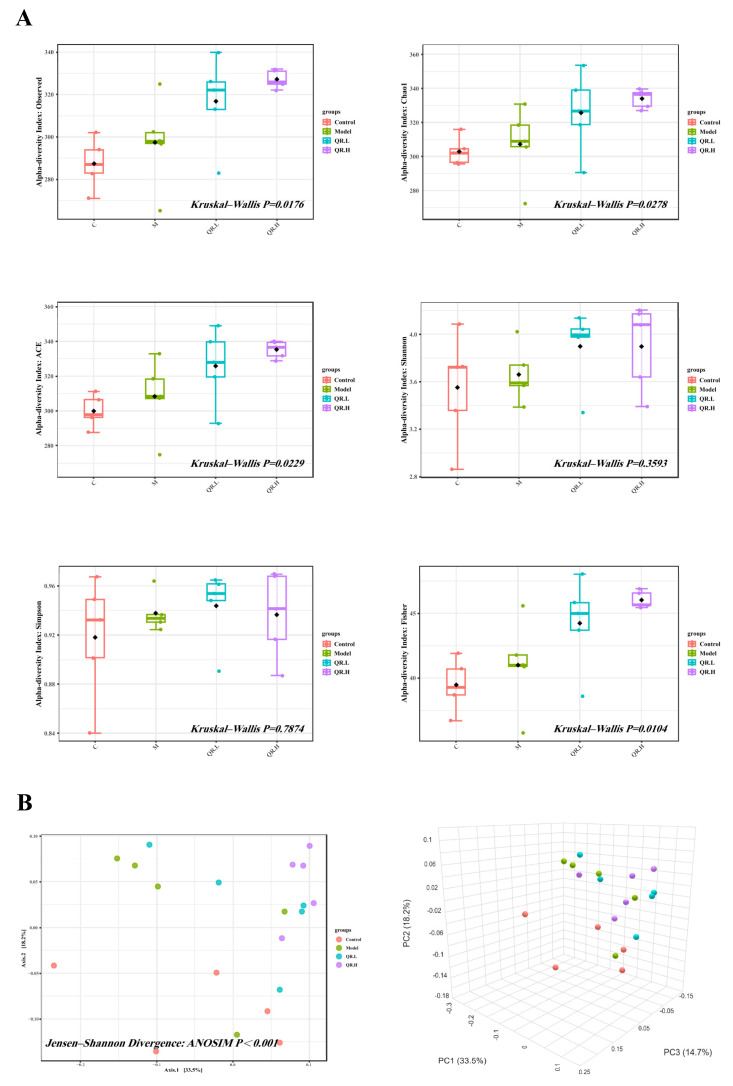
The diversity analysis. (**A**) Alpha diversity indices (observed species, Chao1 index, ACE index, Shannon index, Simpson index, and Fisher index) of the gut microbial communities in the feces (*n* = 5/group). (**B**) Beta diversity (*n* = 5/group). PCoA was used to assess beta diversity.

The microbial community richness indicated by ACE and the Chao1 were significantly different among the four groups (*p* < 0.05, Figure 4A). Additionally, an obviously different ‘observed’ estimator of the four groups implied variations in the total number of features per sample (*p* < 0.05, Figure 4A). Finally, there was noticeable difference in the ‘Fisher’ index, which modeled the community abundance structure as a logarithmic series distribution (*p* < 0.05, Figure 4A). The β diversity was analyzed by PCoA to compare the microbial community and composition between the four groups. There were statistically significant differences in the overall composition of the gut microbiota at the OTU level between the four groups (*p* < 0.001, Figure 4B).

**Figure 5:**
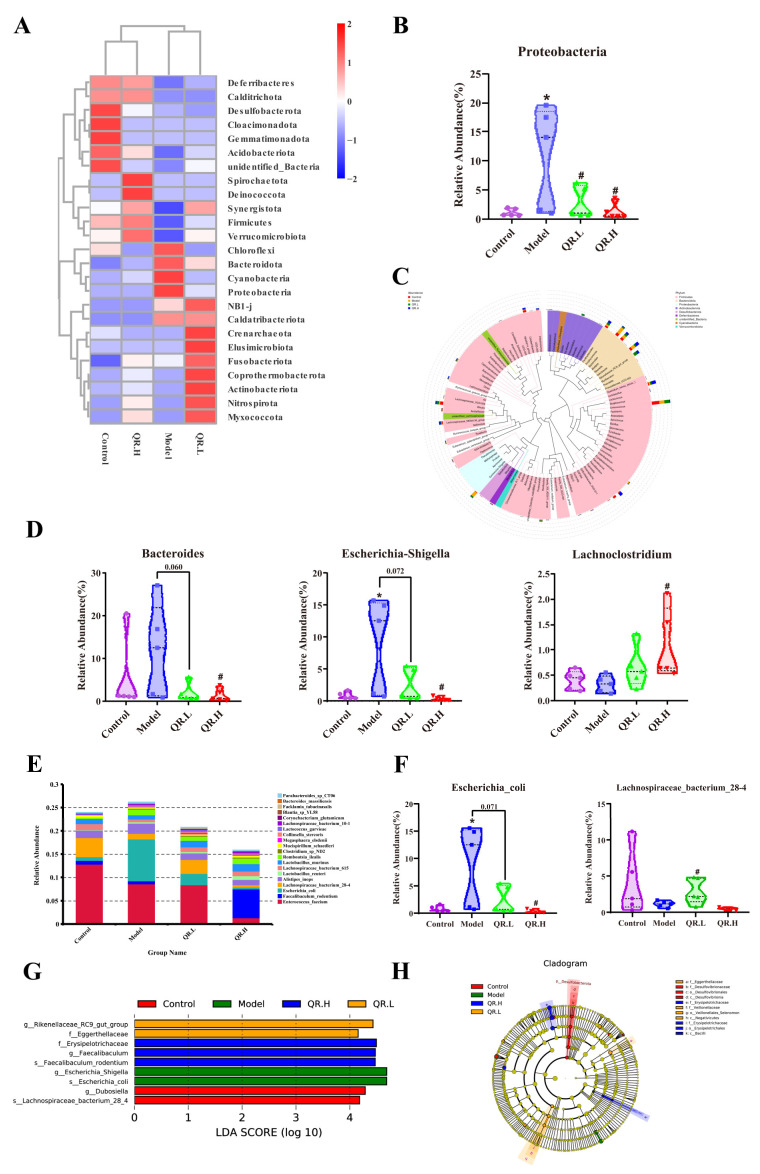
Effects of quercetin on gut microbiota in *db*/*db* mice after 10 weeks of treatment. (**A**) Clustering heat map of relative abundance at phylum level (top 25). (**B**) The relative abundance of Proteobacteria. (**C**) Evolutionary tree at the genus level. (**D**) The relative abundance of *Bacteroides*, *Escherichia-Shigella,* and *Lachnoclostridium*. (**E**) The relative abundance of microbiota at species level (top 20). (**F**) The relative abundance of *Escherichia_coli* and *Lachnospiraceae_bacterium_28-4*. (**G**) Histogram of LDA value distribution. Microbiota with an LDA score greater than 4 were shown in the histogram. The length of the bars represents the effect size of the differing species. (**H**) Cladogram generated from LEfSe analysis. Data are expressed as means ± SEM (*n* = 5/group). * *p* < 0.05 vs. control; ^#^ *p* < 0.05 vs. model.

After Silva database annotation of OTU sequences, the relative abundance of intestinal flora at different taxonomic levels was calculated and compared between different groups. At the phylum level, Firmicutes, Bacteroidota, Proteobacteria, and Actinobacteriota were the common dominant phyla in four groups (Figure 5A). Apparently, the relative abundance of Proteobacteria in diabetic mice increased by 9.60% (*p* < 0.05). Subsequently, compared with the model group, quercetin treatment reduced the relative abundance of Proteobacteria in the QR.L and QR.H groups by 7.95% and 9.52% (*p* < 0.05), respectively (Figure 5B).

At the genus level, four commonly dominant genera were identified, *Enterococcus* and *Lactobacillus,* belonging to the phylum Firmicutes, and *Bacteroides* and *Odoribacter*, belonging to the phylum Bacteroidota (Figure 5C). Compared with the control group, the abundances of *Bacteroides*, *Alistipes*, *Odoribacter*, *Escherichia-Shigella*, *Erysipelatoclostridium*, and *Romboutsia* in the model group were higher, accompanied by lower abundances of *Intestinimonas*, *Oscillibacter*, *Parabacteroides*, and *Lachnoclostridium* (Figure 5C). A total of 0.2% quercetin treatment significantly decreased the relative abundance of the *Bacteroides* and *Escherichia-Shigella* by 10.43% and 8.67%, respectively (*p* < 0.05), while significantly increasing *Lachnoclostridium* by 0.77% in comparison with the model group (*p* < 0.05) (Figure 5D).

The relative abundance of the top 20 species was also calculated (Figure 5E). Compared with control group, the relative abundance of *Escherichia_coli* was significantly increased in *db*/*db* mice. Furthermore, the relative abundance of *Escherichia_coli* was markedly reduced in the OR.L group (*p* = 0.071) and QR.H group (*p* < 0.05) (Figure 5F). Though there was no significant difference in the relative abundance of *Lachnospiraceae_bacterium_28-4* between the control and the model group, its abundance was significantly increased in the QR.L group (*p* < 0.05) (Figure 5F).

The linear discriminant analysis (LDA) effect size (LEfSe) was conducted to further excavate potential bacterial biomarkers among the groups (LDA score ≥ 4). Two bacterial markers (*g_Dubosiella* and *s_ Lachnospiraceae_bacterium_28_4*) in the control group, two bacterial markers (*g_ Escherichia_Shigella* and *s_ Escherichia_coli*) in the *db*/*db* mice, two bacterial markers (f_Eggerthellaceae and *g_ Rikenellaceae_RC9_gut_group*) in the QR.L group, and three bacterial markers (f_Erysipelotrichaceae, *g_Faecalibaculum,* and *s_Faecalibaculum_rodentium)* in the QR.H group were discriminated (Figure 5G), and these abundant taxa can be considered potential biomarkers for the corresponding groups (Figure 5H). These results indicated that the quercetin-supplemented diet significantly remodeled the intestinal microbiota compared with the normal diet.

### 3.4. Quercetin Altered the Metabolites of Gut Microbiota in db/db Mice

**Figure 6:**
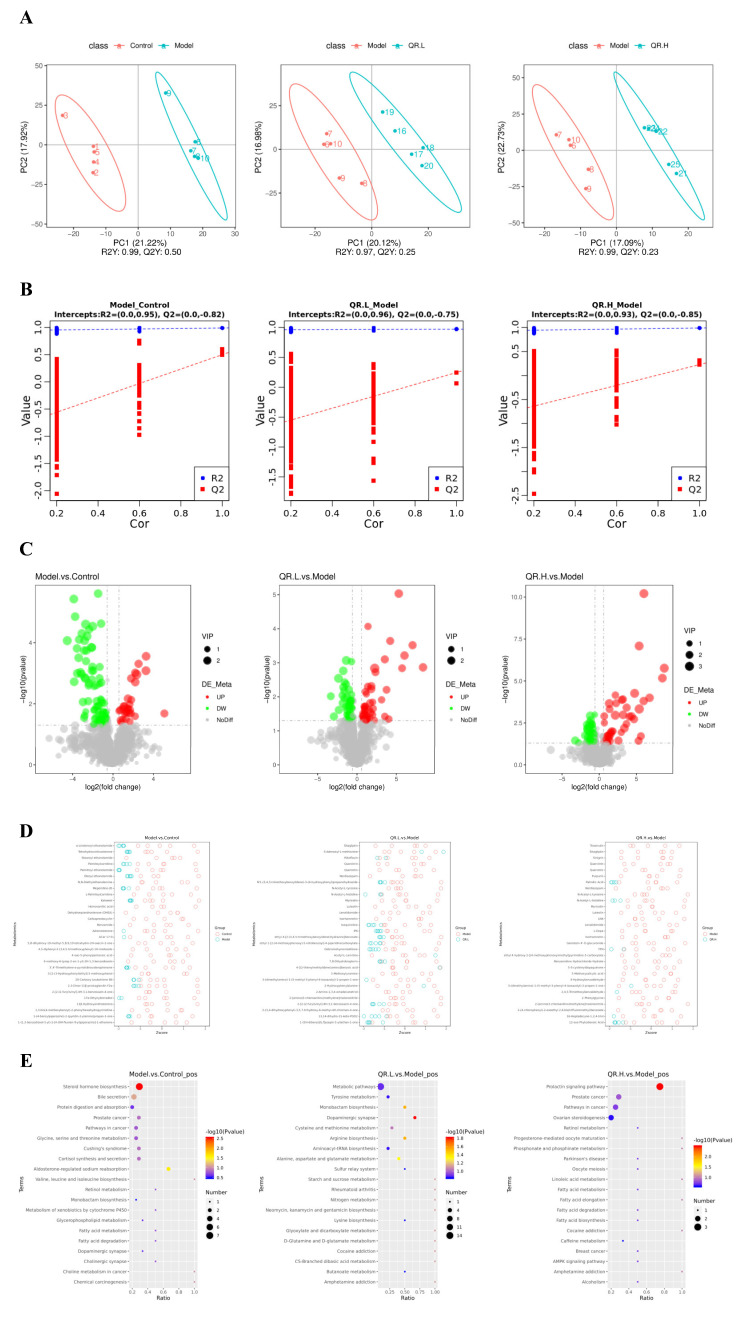
Quercetin altered the metabolites of gut microbiota in *db*/*db* mice. (**A**) PLS-DA scatter diagram. The R2Y metric represents the interpretation rate of the PLS-DA model, while Q2Y evaluates the model’s predictive ability. When R2Y exceeds Q2Y, it indicates the PLS-DA model is well established. (**B**) Sort verification diagram. (**C**) Volcano map. The X-axis indicates the variation in the multiplicity of differences of metabolites among the groups, while the Y-axis indicates the statistical significance level of those differences. (**D**) Z-score analysis. The Z-score, also known as the standard score, is used to quantify the relative content of metabolites on a standardized scale. These figures display the Z-score values for the top 30 metabolites, ranked from smallest to largest *p*-value. (**E**) KEGG enrichment scatterplot.

Firstly, partial least squares discriminant analysis (PLS-DA) was used to establish a model for the relationship between metabolite expression and sample categories. There was a clear trend of separation between the model group and the other three groups (Figure 6A). The model evaluation parameters R2 and Q2 are both close to 1. R2 is greater than Q2 and the intercept of Q2 regression line and Y-axis is less than 0, indicating that the model is stable and reliable (Figure 6B). The volcano plots visually showed the overall distribution of differential metabolites (Figure 6C). In the model group, 30 metabolites were significantly upregulated, while 81 metabolites were significantly downregulated. Compared with the model mice, 49 metabolites were upregulated and 44 were downregulated in the QR.L group, and 44 were upregulated and 44 were downregulated in the QR.H group (Figure 6C). Z-score is a value converted based on the relative content of metabolites, and the top 30 metabolites in the two groups are shown in Figure 6D.

Further, we performed KEGG enrichment analyses to explore the functional pathways involved in the differentially expressed metabolites. The results demonstrated that there were two metabolic pathways, including steroid hormone biosynthesis and aldosterone-regulated sodium reabsorption, enriched in the model group (*p* < 0.05) (Figure 6E). Then five metabolic pathways were selected as potentially enriched pathways for quercetin intervention in T2DM, including the prolactin signaling pathway, the dopaminergic synapse, arginine biosynthesis, monobactam biosynthesis, and alanine, aspartate, and glutamate metabolism (*p* < 0.05) (Figure 6E). The differential metabolites of the four groups were determined according to a variable importance of projection (VIP) value >1 and *p* < 0.05. Based on this qualification, we found that 12 metabolites showed significant changes in these metabolic pathways (Table 1). The levels of L-Dopa and S-Adenosyl-L-methionine (SAM) were significantly increased, but 3-Methoxytyramine (3-MET), L-Aspartic acid, and L-Glutamic acid were significantly decreased in the QR.L group. On the other hand, the level of L-Dopa was inclined to increase, but Androstenedione was decreased in the QR.H group. Among these metabolites, L-Dopa was involved in the dopaminergic synapse and prolactin signaling pathway. Moreover, L-Aspartic acid was involved in arginine biosynthesis, monobactam biosynthesis, and the alanine, aspartate and glutamate metabolism pathway, and L-Glutamic acid was involved in arginine biosynthesis and the alanine, aspartate, and glutamate metabolism pathway (Table 1).

### 3.5. Potential Association of Gut Microbiome and Biochemical Indicators with Metabolites

**Figure 7:**
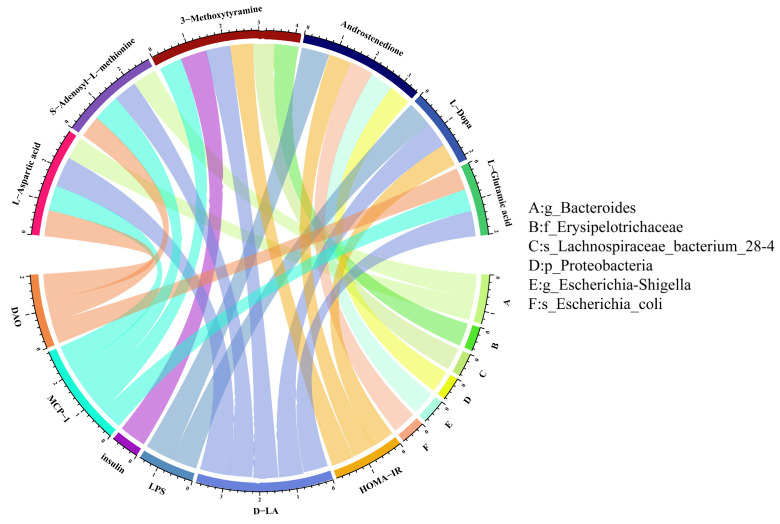
Association of gut microbiome and biochemical indicators with metabolites. The metabolites are on the underside, and the biochemical indicators and gut microbiome are on the upside.

To investigate the phenotypic changes that may be caused by the alteration of the microbial community structure, we performed Pearson correlation analysis of significantly different metabolites obtained from metabolomics analysis with different gut microbiota and significantly different biochemical indicators. As shown in Figure 7, *g*_*Bacteroides* was positively correlated with L-aspartic acid but negatively with SAM. In addition, 3-MET was positively correlated with f_Erysipelotrichaceae and negatively related to *s*_*Lachnospiraceae_bacterium_28-4*. Furthermore, we found that there was a positive correlation between androstenedione and the relative abundance of *p*_Proteobacteria, *g*_*Escherichia-Shigella,* and *s*_*Escherichia_coli*. We further analyzed the association between metabolites and biochemical indicators. The results showed that 3-MET was positively associated with the levels of insulin, HOMA-IR, MCP-1, and D-LA. Both L-Glutamic acid and L-Aspartic acid were positively correlated with MCP-1, DAO, and D-LA, but SAM was negatively related to the levels of MCP-1, DAO, and D-LA. Also, Androstenedione was positively associated with HOMA-IR and LPS. Finally, L-Dopa was inversely related to the levels of HOMA-IR, D-LA, and LPS (Figure 7).

## 4. Discussion

In this study, we assessed the effects of quercetin addition on T2DM mice. The *db*/*db* mice adopted in this study have been recognized as a spontaneous model of T2DM and widely used to investigate pathogenesis and complications of diabetes [26,27]. The *db*/*db* mice exhibit significant symptoms of diabetes at four weeks of age and develop severe hyperglycemia after eight weeks. Extensive research has demonstrated that quercetin is effective in the treatment of T2DM [28,29,30]. It has been shown that quercetin can efficiently alleviate early diabetic renal injuries and decreased blood glucose [31]. Our results showed that quercetin significantly reduced FBG levels in the first three weeks of the experiment, which suggested quercetin possessed the therapeutical effect on the early stage of diabetes. Meanwhile, the body weight of *db*/*db* mice treated with 0.1% quercetin decreased at the later stage of the experiment (after the seventh week), and the feed consumptions of the quercetin-treatment groups, especially the QR.L group, were lower than that of the model group, which indicated that quercetin could alleviate polyphagia and obesity in *db*/*db* mice. The above results prove that quercetin intervention can exert anti-diabetic effects in *db*/*db* mice.

T2DM is characterized by insulin resistance, which is manifested by decreased sensitivity to insulin in target tissues, such as the liver [32]. Our results indicated that quercetin could reduce serum insulin and MCP-1 levels in db/db mice. As a result, T2DM is commonly accompanied by elevated insulin levels in the body. We found that supplementation with quercetin in the AIN-93M diet significantly decreased insulin and HOMA-IR in *db*/*db* mice, indicating that quercetin may have an effect on improving insulin resistance. The pro-inflammatory factor MCP-1 plays an important mediation role in diabetic complications, such as diabetic retinopathy and diabetic nephropathy (DN) [33,34]. It has been shown that suppressing MCP-1 can improve inflammation in *db*/*db* mice [35]. Research from the past few years has shown that kidney damage in DN can be ameliorated by inhibiting macrophage infiltration via blocking the MCP-1/CCR2 pathway [36,37]. Our result demonstrated that quercetin greatly decreased the level of serum MCP-1 in *db*/*db* mice.

A growing body of clinical evidence has found that there are disturbances of the intestinal microbiota and increased intestinal permeability in T2DM patients [12,38]. The levels of DAO, D-LA, and LPS in the serum are biomarkers of the intestinal permeability. DAO is an intracellular enzyme in the intestinal mucosa and epithelium. D-LA and LPS are metabolites of bacteria inherent in the intestinal canal. When the intestinal barrier function is damaged, a large amount of D-LA, DAO and LPS enter the blood circulation through the damaged intestinal mucosa [39,40]. Quercetin has been reported to protect against intestinal barrier disruption caused by acute necrotizing pancreatitis [41]. In the present study, quercetin treatment significantly reduced the levels of serum D-LA, DAO, and LPS. These results demonstrate that the intestinal permeability of *db*/*db* mice was significantly destroyed and that quercetin was effective in repairing intestinal barrier dysfunction, which in turn improved the symptoms of diabetes.

T2DM is associated with changes in the structure and homeostasis of the gut microbiota. Disturbances in the intestinal microbiota and destruction of the intestinal barrier can impair the organs of T2DM patients [14]. A population-based cross-sectional study revealed that gut microbiota composition was altered both in prediabetes and T2DM groups [13]. Another cross-sectional study found that higher microbiome alpha diversity was related to less T2DM [42]. Polyphenolic compounds have been found to have effects on the regulation of intestinal microbiota, with quercetin being the main component at work [43,44,45]. Quercetin can improve microbial community diversity and increase intestinal probiotic levels in type 2 diabetic mice [21]. In the present study, the observed, Chao1, ACE and Fisher indices were significantly increased in quercetin-treatment groups than those of the model group, indicating that quercetin remarkably improved the microbiota diversity and homogeneity in diabetic mice.

It is well known that Firmicutes, Bacteroidota, Proteobacteria, and Actinobacteriota are the dominant phyla in the intestinal canal [46]. The relative abundance of Proteobacteria in the gut microbiota was significantly higher in patients with T2DM [47,48,49]. Several works have suggested that the Proteobacteria level is related to insulin resistance and that depleting Proteobacteria attenuates systemic inflammation and insulin resistance [50,51]. Consistent with previous studies, our result showed that the relative abundance of Proteobacteria in diabetic mice was reduced significantly after quercetin (0.1% and 0.2%) treatment. At the genus level, we found that the relative abundance of *Bacteroides*, *Escherichia-Shigella,* and *Lachnoclostridium* was significantly different among the groups. A clinical study revealed that the abundance of *Bacteroides*, especially *Bacteroides fragilis,* was reduced in T2DM patients after metformin treatment, which contributed to an improvement in glucose intolerance by metformin [52]. Similarly, another study showed that Acarbose reduced *Bacteroides* levels in diabetic patients [53]. After three weeks of metformin intervention, the abundance of *Lachnoclostridium* in feces of diabetic patients significantly increased [54]. A growing body of evidence now suggests that *Escherichia-Shigella* is closely associated with T2DM. The abundance of *Escherichia-Shigella* was increased in individuals with T2DM [54,55] and was positively related with the FBG level [55]. Animal experiment also revealed that the abundance of *Escherichia-Shigella* was increased in rats with T2DM and positively correlated with FBG, HOMA-IR, and LPS level [56]. *Escherichia-Shigella* might be a new potential biomarker of diabetic nephropathy (DN) [57]. *Escherichia coli* is a subspecies of *Escherichia-Shigella*. Persistent *Escherichia coli* asymptomatic bacteriuria is a common occurrence in patients with diabetes [58]. Our results demonstrate that *Escherichia coli* follows the same trend as *Escherichia-Shigella,* suggesting that *Escherichia coli* may be the species playing the main role.

To be specific, we found that quercetin may affect the phenotype of diabetes through five metabolic pathways. KEGG pathway analysis showed the up-regulated L-Dopa was enriched in the dopaminergic synapse and prolactin signaling pathway. Some studies using network pharmacological analysis and molecular docking validation found that the prolactin signaling pathway was closely related to the hypoglycemic mechanism [59,60]. L-Dopa is a precursor of dopamine (DA), which crosses the blood–brain barrier and is converted to DA by the action of dopa decarboxylase to exert its pharmacological effects. Some studies have revealed that dopamine has an effect on glucose homeostasis and pancreatic *β*-cell function [61]. Quercetin intervention upregulated L-Dopa, suggesting that L-Dopa may influence the phenotype of diabetes (such as HOMA-IR) via the dopaminergic synapse and prolactin signaling pathway. There are few studies on the relationship between 3-MET and diabetes. The role of 3-MET, a factor in the dopaminergic synapse pathway, may be similar to that of L-Dopa. A European prospective investigation found that the concentration of androstenedione was lower in men with diabetes compared to men without diabetes [62]. The contribution of 3-MET and androstenedione in type 2 diabetes still needs to be further explored. Similarly, our association analysis found that L-Dopa, androstenedione, and 3-MET were associated with insulin and HOMA levels. On the other hand, Proteobacteria, *Escherichia-Shigella*, *Escherichia_coli*, and *Lachnospiraceae_bacterium_28-4* were associated with the above three metabolites, suggesting that quercetin may regulate glucose metabolism in diabetic mice through the microbiota.

Previous studies have provided evidence that insulin secretion can be regulated by certain specific amino acids [63]. Our metabolomics analysis also identified a number of differential amino acids in the feces. L-aspartic acid and L-glutamic acid were both enriched in arginine biosynthesis, and the alanine, aspartate, and glutamate metabolism pathway. In addition to this, L-aspartate and SAM were involved in the monobactam biosynthesis pathway. Metabolomics analysis of DN patients in previous studies also identified arginine biosynthesis and the alanine, aspartate, and glutamate metabolism pathway [64]. L-glutamic acid is abundant in the body, is involved in a variety of amino acid metabolisms, and regulates a number of important physiological metabolic processes. There was a study that performed a metabolomic analysis of mice feces and found increased levels of L-aspartic acid, which have been associated with lipogenesis and inflammation, in the feces of prediabetic mice compared to normal mice [65]. L-glutamic acid may be a potential biomarker of diabetic retinopathy [66,67] and act as an amplifying signal in insulin secretion induced by incretin [68]. A clinical study found that diabetic patients, especially those with diabetic nephropathy, presented with abnormal concentrations of SAM and its related compounds in the blood. The high mortality and morbidity in diabetic nephropathy patients may be associated with methyl deficiency due to SAM deficiency [69]. In addition to this, our study found that these metabolites were associated with indicators of intestinal barrier function, such as DAO and D-LA, and *Bacteroides* was related to L-aspartic acid and SAM. Quercetin may repair intestinal barrier function by regulating the abundance of *Bacteroides*.

Quercetin and its derivatives are flavonoid compounds derived from natural plant sources, with wide availability and relatively low toxicity. Their application in the clinical treatment of diabetes can alleviate adverse reactions in patients and significantly reduce treatment costs. The low and high doses (0.1% and 0.2%) selected in this study correspond to human equivalent doses of 961.5 mg and 1923 mg, respectively (based on a 70 kg adult body weight). However, intervention studies in patients with T2DM are still needed to determine the effects of quercetin and the appropriate intervention dosage. Therefore, developing diabetes nutritional supplements with quercetin as the primary functional ingredient and deeply exploring the mechanisms by which quercetin can prevent and treat T2DM hold great social value and scientific significance.

## 5. Conclusions

This study proposed the gut microbiome as the target of action and investigated the potential mechanisms by which quercetin improved the symptoms of T2DM within an animal model. Our results indicated that the supplementation of quercetin to the AIN-93M diet reduced body weight, alleviated FBG on initial stage of T2DM, and decreased the levels of serum insulin and HOMA-IR in *db*/*db* mice. Furthermore, quercetin was also able to reduce the levels of serum MCP-1 and intestinal permeability biomarkers (DLA and LPS). Sequencing of 16S rRNA genes demonstrated that quercetin could significantly decrease the abundance of potentially detrimental bacteria, such as Proteobacteria, Proteobacteria, *Bacteroides*, *Escherichia-Shigella*, and *Escherichia_coli*. Moreover, untargeted metabolomics analysis revealed that quercetin intervention altered the metabolites of *db*/*db* mice, resulting in significant increases in L-Dopa and SAM levels but significant decreases in 3-MET, L-Aspartic acid, L-Glutamic acid, and androstenedione levels. In summary, quercetin has a good anti-diabetic effect on *db*/*db* mice by alleviating insulin resistance, repairing intestinal barrier, remodeling the intestinal microbiota, and altering the metabolites.

## Figures and Tables

**Table 1:** Differential metabolites and corresponding metabolic pathways between groups.

Metabolic Pathway	Metabolites	Formula	VIP	Trend
Model vs. control				
Steroid hormone biosynthesis				
	Tetrahydrocorticosterone	C_21_H_34_O_4_	2.38	down
	Cortisol	C_21_H_30_O_5_	1.52	down
	Adrenosterone	C_19_H_24_O_3_	2.03	down
	Etiocholanolone	C_19_H_30_O_2_	2.03	up
	16α-Hydroxyestrone	C_18_H_22_O_3_	1.81	down
	Estriol	C_18_H_24_O_3_	2.15	down
Aldosterone-regulated sodium reabsorption				
	Cortisol	C_21_H_30_O_5_	1.52	down
QR.L vs. Model				
Dopaminergic synapse				
	L-Dopa	C_9_H_11_NO_4_	1.46	up
	3-Methoxytyramine (3-MET)	C_9_H_13_NO_2_	1.69	down
Arginine biosynthesis				
	L-Aspartic acid	C_4_H_7_NO_4_	1.80	down
	L-Glutamic acid	C_5_H_9_NO_4_	1.53	down
Monobactam biosynthesis				
	L-Aspartic acid	C_4_H_7_NO_4_	1.80	down
	S-Adenosyl-L-methionine (SAM)	C_15_H_22_N_6_ O_5_S	1.80	up
Alanine, aspartate and glutamate metabolism				
	L-Aspartic acid	C_4_H_7_NO_4_	1.80	down
	L-Glutamic acid	C_5_H_9_NO_4_	1.53	down
QR.H vs. model				
Prolactin signaling pathway				
	L-Dopa	C_9_H_11_NO_4_	1.83	up
	Androstenedione	C_19_H_26_O_2_	2.12	down

## Data Availability

The data presented in this study are available on request from the corresponding author.

## Supplementary Materials

The following supporting information can be downloaded at https://www.mdpi.com/article/10.3390/nu16121870/s1, Figure S1: (A) Rarefaction curve. (B) Rank abundance curve. (C) Species accumulation boxplot. When the boxplot distribution appears relatively flat, it suggests the species richness does not increase substantially with larger sample sizes, indicating the data are suitable for further analysis.
