# Supplementary material for: Quercetin Alleviates Insulin Resistance and Repairs Intestinal Barrier in db/db Mice by Modulating Gut Microbiota
Source: Nutrients. 2024 Jun 14;16(12):1870. doi: 10.3390/nu16121870 (PMC11206920; doi:10.3390/nu16121870)
Supplement: Supplementary file 1 [file nutrients-16-01870-s001.zip › nutrients-3038013-supplementary.pdf]

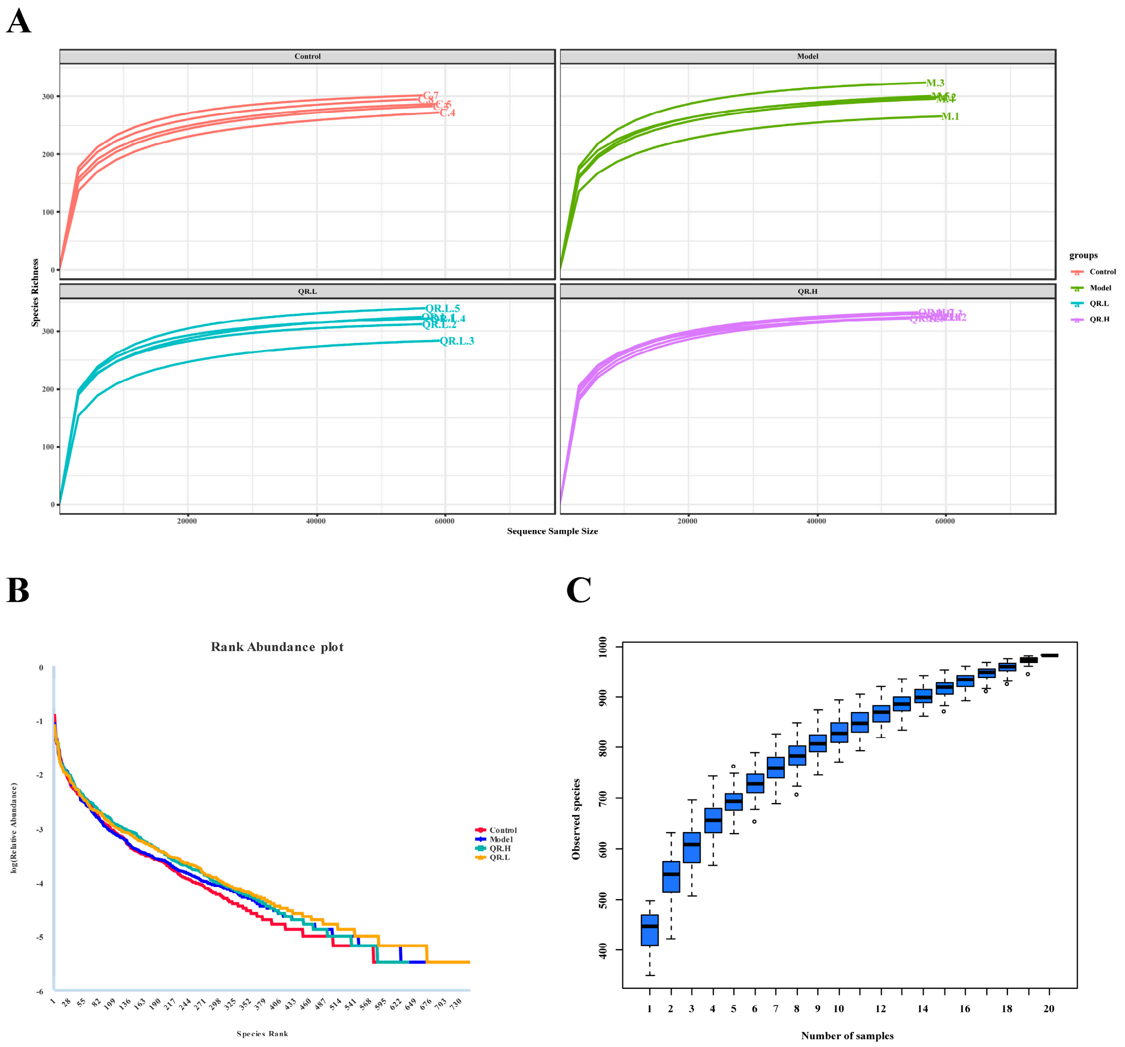

Figure S1: (A) Rarefaction curve. (B) Rank abundance curve. (C) Species accumulation boxplot. When the boxplot distribution appears relatively flat, it suggests the species richness does not increase substantially with larger sample sizes, indicating the data are suitable for further analysis.
